# Elastic evolution of a self-healing ionomer observed via acoustic and ultrasonic resonant spectroscopy

**DOI:** 10.1038/s41598-017-14321-z

**Published:** 2017-10-31

**Authors:** K. A. Pestka, J. D. Buckley, S. J. Kalista, N. R. Bowers

**Affiliations:** 1 Department of Chemistry and Physics, Longwood University, Farmville, Virginia 23909 USA; 2 Department of Biomedical Engineering, Rensselaer Polytechnic Institute, Troy New York, 12180 USA; 3 Department of Physics, Rollins College, Winter Park, Florida 32789 USA

## Abstract

Self-healing poly (ethylene co-methacrylic acid) ionomers (EMAA) are thermoplastic materials that when punctured, cut, shot or damaged in a variety of ways, are capable of autonomously reorganizing their physical structure to heal and, in many instances, permanently seal the damaged location. However, a complete picture of the mechanisms responsible for their unusual behavior is not well understood. In this article we report the observation of time dependent acoustic and ultrasonic spectral evolution, measured using resonant acoustic and ultrasonic spectroscopy, for both pre and post-damage EMAA samples. The results provide a means to differentiate healing phases, quantify healing timescales, and potentially elucidate the composition parameters that most significantly impact healing behavior.

## Introduction

Poly (ethylene co-methacrylic acid) ionomers (EMAA) are thermoplastic materials that are capable of autonomously self-healing and have potential applications such as emergency containment, self-healing consumer products and others^1–3^. However, unlike many other self-healing designs that require micro-encapsulation or other additives activated during the damage and healing phases^4–7^, EMAA samples can be fabricated as single component autonomous self-healing films and structures. Relatively recent experiments have focused on testing EMAA samples via Raman spectroscopy, ballistic impact and thermal variation^8–10^. Those previous experiments are consistent with three distinct timescales that appear during the healing process. The first is an elastic melt state, instantly after damage, where the healing process is dominated by energy transfer from the damage source producing molten material flow, followed by a secondary, short term welding and solidification phase, and a tertiary long-term evolving polymer interdiffusion. Those prior experiments are also consistent with secondary and tertiary healing phases dominated by polymer chains that form domains and reorient during and after damage. It has also been proposed that the efficacy of the healing mechanism may be affected by the molecular weight, ionic content as well as sample temperature, sample age, preparation conditions and damage type; however, a complete picture of the healing behavior is still not fully understood.

Recently, efforts to improve characterization of these unique materials led to the use of Time Dependent Resonant Spectroscopy (TDRS) as a means to assess spectral variation in post-damage EMAA samples^11^. The resonant spectral and vibrational properties of a system can be determined, in principle, from sample geometry, density and elastic properties. Vibrational resonant techniques are an effective probe of the atomic and molecular environment and have enabled the characterization of a wide range of materials, and microstructures in single-crystal, thin-film and other forms^12–16^. Vibrational resonant techniques are also applicable to macroscopic systems such as cars, planes, buildings, bridges, as well as planets and stars^17–19^. Ultrasonic resonant techniques have been especially well suited to study phase transitions including superconductivity and others, especially when the environmental parameters such as temperature and magnetic field can be controlled^20–26^. Recent experimental methods have also extended acoustic and ultrasonic resonant techniques to characterization of visco-elastic and polymeric materials^11,27^. Historically however, resonant acoustic and ultrasonic methods, while capable of assessing the effects of sample aging^28^, were not well suited to observe real-time phase transitions caused by physical damage due to the extremely rapid relaxation times in common materials. However, due to the unusual properties of self-healing EMAA polymers, TDRS was used recently to discover resonant spectral transitions in post-damage samples during the time period immediately after macroscopic structural changes were no longer visible^11^.

The discovery of spectral variation in damaged EMAA samples led to an increased interest in acoustic methods as a means to determine healing timescales and improve sample characterization. Building on that work, we used TDRS to observe post-damage resonant spectral shifts and determine the approximate secondary and tertiary post-damage healing timescales of an EMAA ionomer. In addition, it was discovered that persistent spectral evolution was present in all samples for both undamaged EMAA samples as well damaged EMAA samples after their post-tertiary healing phase was complete.

## Experimental Methods and Results

Determination of the spectral evolution was accomplished by measuring the acoustic and ultrasonic spectra of both pre and post-damage EMAA samples. The results reported in this article are from three samples of EMAA-0.6Na, known as DuPont Surlyn 8920, with 60% of the methacrylic acid groups neutralized by sodium. Samples were cut into parallelepiped shapes from an approximately 1.4mm thick EMAA pressed film using a utility razor. Details of the EMAA pressed film production can be found in the literature^11^. Data from the three identically prepared samples reported here had dimension (3.56 ± *0*.*04*) × (4.64 ± *0*.*05*) × (1.464 ± *0*.*002*) mm^3^, (*7*.*11* ± *0*.*04*) × *(7.86* ± 0.07) × (*1*.*43* ± *0*.*01*) mm^3^ and (4.23 ± *0*.*09*) × (4.11 ± *0*.*02*) × (1.33 ± *0*.*01*) mm^3^, hereafter called *samples a*, *b* and *c* respectively. Prior to spectral measurement, all samples were stored at room temperature in hermetically sealed containers with desiccant in order to minimize moisture exposure.

**Figure 1:**
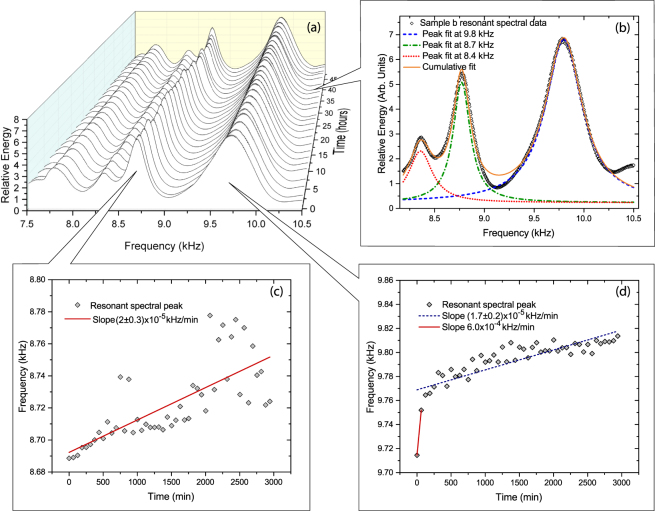
Undamaged Sample Behavior: Time dependence of the partial resonant spectrum of *sample b* over 48 hours (**a**). Lorentzian multi-peak fit to the signal used to extract individual resonances (**b**). Time evolution of the resonant frequencies at approximately 8.7 kHz (**c**) and 9.8 kHz (**d**) for the undamaged EMAA sample.

The resonant spectra of all EMAA samples were measured using a Magnaflux RUSpec resonant spectral system, details of which can be found in the literature^11,29^. Each sample was mounted between two piezo transducers and a range of frequencies, typically 5–50 kHz, were swept. Prior to damage all samples were repeatedly scanned without removal from the RUSpec system over a period of up to 48 hours, in order to provide a baseline for subsequent comparison with damaged samples. Results for *sample b* are shown in Fig. 1. These polymers dissipate their vibrational energy rapidly resulting in resonances that are broad and often overlap, which can complicate the analysis. By using a multi-peak fitting algorithm it was possible to extract the individual resonances, as shown in Fig. 1(b) ^30^.

As can be seen in Fig. 1(c) and (d), it was discovered that the undamaged samples exhibited low-level spectral evolution. In addition, the observed elastic variation was persistent and appeared in all tested EMAA samples with an average value of (*7*.*5* ± *2*) × 10^−*4*^ *kHz/min*. In contrast, spectral measurement of typical high quality crystalline materials studied using an identical experimental configuration, such as samples composed of aluminum, steel, Si, rare earth scandates and others, have not exhibited detectable frequency drift within the experimental resolution of a few parts per million^29^. However, since the RUSpec system was not humidity controlled, sample uptake of moisture is possible during the experiment. In this case the observed low-level spectral and elastic evolution is consistent with the hygroscopic nature of EMAA-0.6NA, with air and moisture exposure affecting sample resonant spectra over time^31^. In addition, other recent experiments have shown that EMAA samples of similar composition become brittle with age, consistent with the elastic evolution reported here^32^.

**Figure 2:**
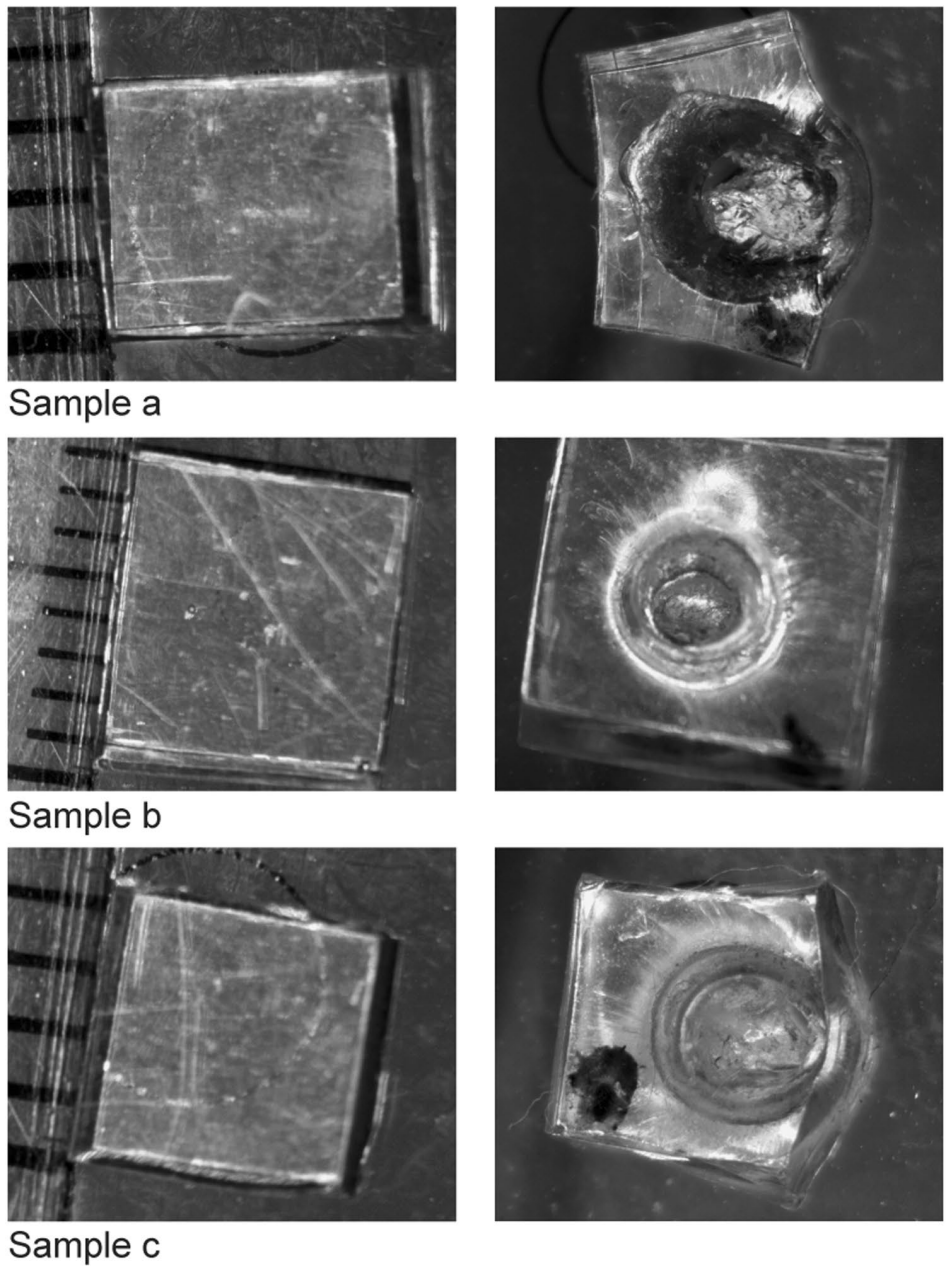
Three EMAA self-healing samples are shown before damage (left) and after damage (right) from a 3mm pin punch. The initial molten state of the EMAA material caused the samples to heal around the 3mm pin punch immediately after impact, leaving behind the cavity shown in the figures. The black mark on each sample was used to preserve orientation during remounting the sample in the RUSpec chamber.

The undamaged samples were then removed from the RUSpec system and damaged using a 3 mm Bostitch pin punch driven into the samples using a 300 gram mass-loaded Dytran 5805 A impulse hammer resulting in significant damage to the samples as shown in the Fig. 2.

Damaged samples were then returned to the RUSpec system as quickly as possible. The time from impact to initiating the spectral scans varied from a minimum of 23 seconds for *sample a* up to a maximum of 157 seconds for *sample b*. The time difference in loading the samples was due in part to samples healing around the pin punch.

**Figure 3:**
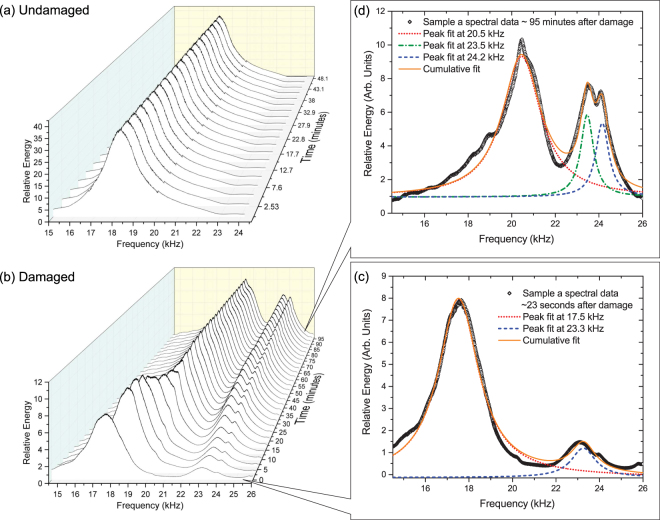
Partial time dependent spectrum of *sample a* before damage (**a**) and after damage (**b**). The lorentzian multi-peak fits are shown just after damage (**c**) and over an hour after damage (**d**).

The resonant spectra of the damaged samples were then repeatedly measured over a period of up to 22 hours without sample removal from the RUSpec chamber. Since the physical geometry is altered during the damage event, the location of the resonant peaks can vary significantly from that of the undamaged samples as can be seen in Fig. 3. However, the change to the resonant spectrum due to the new geometry occurs almost instantly after damage during the first healing phase, typically on the order of less than 1 second. All post-damage spectral evolution reported in this article occurred after this time, where all macroscopic changes in sample geometry had ceased to be visible. As can be seen in Fig. 3(b), dramatic evolution appears in the post-damage resonant spectra after this time.

**Figure 4:**
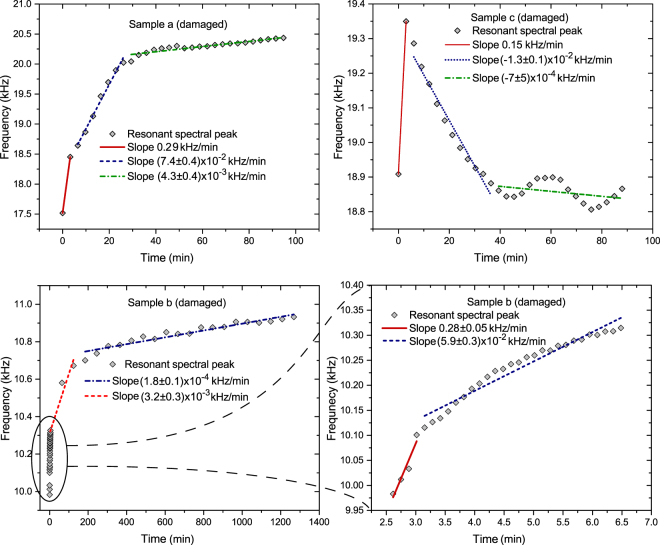
Post-damage resonant spectral analysis for *samples a*, *b* and *c*. All samples exhibit significant change in peak frequencies versus time and eventually return to pre-damage evolutionary rates. Time evolution during the first 100 minutes is shown for *sample a* and *sample c* (top). Time evolution is shown for *sample b* over 20 hours (bottom left) and with increased resolution during the first few minutes after damage (bottom right). Note: all samples exhibit strong elastic stiffening during the first few moments after damage.

Once the spectrum of each post-damage sample was measured, the identified resonant frequencies were plotted as a function of time. Partial spectra illustrating the time dependence for the three different post-damage samples are shown in Fig. 4. As can be seen in Fig. 4, all samples exhibited frequency changes that increased with time and lasted for approximately 3–5 minutes after damage, which typifies the secondary healing phase. For the samples reported here the average rate was (*1*.*9* ± *0*.*6*) × 10^*−1*^ *kHz/min*, approximately two orders of magnitude larger than the undamaged sample rates. After this secondary post-damage state, a transition is visible in all the spectral evolution plots. During the next roughly 30 to 60 minutes, resonant frequency variation continued to occur during the tertiary healing phase, but at a reduced rate of (*4* ± *2*) × 10^*−2*^ *kHz/min*, approximately one order of magnitude larger than the undamaged sample rates. During this phase most, but not all (e.g. *sample c* of Fig. 4), transitions resulted in increased resonant frequency. It is important to note that each resonant mode can couple to different elastic moduli. In the case of sample c, the illustrated mode appears to couple to elastic moduli that experience significant elastic softening during the tertiary healing phase.

After approximately 30–60 minutes all samples returned to an average rate of spectral evolution of (*1*.*9* ± *0*.*9*) × 10^*−3*^ *kHz/min*, which is their approximate pre-damage rates. Prior experiments reported elsewhere estimated that the long-term post-damage elastic evolution of similar EMAA samples may take over 18 hours to reach equilibrium^11^. However, based on data from the long-term post-damage phase and the evolution discovered in undamaged samples reported here, that result can be interpreted as a manifestation of the persistent low-level elastic evolution that is always present in this type of EMAA sample.

In addition, the relative impact force, as measured using the impulse hammer, for *samples b* and *c* were 82% and 50% respectively relative to *sample a*. Also, the total volume of *samples a* and *c* were approximately 30% of that of *sample b*. Thus the observed elastic evolution and healing timescales, which were similar for all tested samples, especially during the secondary phase, do not appear to be significantly influenced by variation in the sample size or impact force. In contrast, the consistent nature of the healing timescales and associated transitions do correlate with the size of the damaged area, which was identical for all samples.

A summary of the healing phases and the approximate timescales using the dominant resonant frequency of each post-damage sample is shown in Table 1.

**Table 1:** Approximate healing timescales and spectral evolution rates of the three post-damage EMAA samples.

Post-Damage Healing Phases	Secondary		Tertiary		Return to Pre-Damage	
	Time (min)	Rate (kHz/min)	Time (min)	Rate (kHz/min)	Time (min)	Rate (kHz/min)
*Sample a* (3.56 × 4.64 × 1.464 mm^3^)	4 ± 1.5	0.29	30 ± 1.5	0.074	>30	0.0043
*Sample c* (4.23 × 4.11 × 1.33 mm^3^)	4 ± 1.5	0.15	40 ± 1.5	−0.013	>40	−0.00072
*Sample b* (7.11 × 7.86 × 1.43 mm^3^)†	3 ± 0.13	0.28	60 ± 30	0.059	>60	0.00018

Note: these times include the time from impact to the moment the samples were loaded into the RUSpec chamber. ^†^For *sample b*, the scanning interval was approximately every 8 seconds during the first 7 minutes after damage, then once per hour for the next 22 hours, resulting in improved precision for the secondary healing timescale but reduced precision for the tertiary healing timescale. For the other samples, the scanning interval was approximately 3 minutes.

## Conclusion

In summary, resonant modes whose resonant frequencies change with time were discovered in both undamaged and damaged EMAA samples, indicative of persistent elastic evolution. Immediate post-damage spectral evolution was observed to increase by approximately two orders of magnitude, and in all cases was characterized by an elastic stiffening phase. Afterwards subsequent post-damage elastic variation, both stiffening and softening, continued for the next 30 to 60 minutes, at approximately one order of magnitude above the pre-damaged values.

For the post-damage interval, the magnitude of the spectral evolution rates made it possible to differentiate the elastic evolution into distinct healing phases, consistent with previous experiments: an elastic melt state, instantly after the damage, a secondary, short term welding and solidification phase, and a third long-term phase. Also, the TDRS method, when applied to post-damage EMAA samples, provided a mechanism to quantify the post-damage timescales.

After the tertiary healing phase, spectral evolution decreased to levels comparable to their pre-damage evolutionary rates, indicating persistent elastic evolution is always present in these EMAA samples and that environmental exposure may affect the long-term self-healing efficacy. This attribute may significantly influence the applicability of EMAA self-healing materials for extended exposure applications. Additionally, differences in sample composition are expected to alter the sample resonant spectrum and potentially the associated time-dependent spectral evolution both before and after damage, if so, then it should be possible to use the TDRS technique to quantify and differentiate the influence of EMAA sample composition, such as ionic content, molecular weight and age, as well as sample geometry and damage conditions on the sample elastic variation and overall healing behavior.

### Data Statement

Access to data presented in this work is available upon reasonable request from the corresponding author.
